# Importance of providing pharmaceutical care for patients using psychotropic medicines in community pharmacy

**DOI:** 10.3389/fpubh.2025.1747111

**Published:** 2026-01-12

**Authors:** Alena Tatarević, Monika Popčević, Nataša Bogavac Stanojević, Arijana Meštrović, Lovorka Bilajac

**Affiliations:** 1 Pharmacy Bobanović Vujnović, Pula, Croatia; 2 Pharma Expert d.o.o, Zagreb, Croatia; 3 Faculty of Pharmacy, Belgrade University, Belgrade, Serbia; 4 Faculty of Medicine, University of Rijeka, Rijeka, Croatia; 5 Faculty of Health Studies, University of Rijeka, Rijeka, Croatia; 6 Teaching Institute of Public Health Primorje-Gorski Kotar County, Rijeka, Croatia

**Keywords:** community pharmacists, drug-related problems, mental health, patient safety, pharmaceutical care, psychotropic medicines, public health, quality of life

## Abstract

**Background:**

Mental health represents a major global public health challenge significantly impacting well-being, quality of life, and mortality. Psychotropic medicines are frequently associated with drug-related problems (DRPs) that may lead to decreased quality of life (QoL), falls, hospitalizations, and increased morbidity, or mortality. Non-adherence, observed in nearly half of patients with psychiatric disorders, remains a critical issue. This study evaluated the real-life impact of community pharmacists in identifying and managing DRPs among patients using psychotropic medicines and examines how sociodemographic factors influence QoL.

**Methods:**

The randomized controlled study was conducted in six community pharmacies in the Istrian County (Croatia) among adult patients using psycholeptics or psychoanaleptics. DRPs were documented using the Pharmaceutical Care Network of Europe (PCNE) DRP Classification Version 9.1. QoL was assessed once, prior the intervention, with the WHOQoL-BREF Questionnaire. In the intervention group (A), pharmacists’ intervention followed a standardized protocol, in contrast to common pharmacists’ practice in Croatia provided to the participants in the control group (B).

**Results:**

Ninety-seven participants completed the study. Baseline measurement of QoL showed significantly higher scores in Physical health (*p* = 0.018) and Social interaction (*p* < 0.001) domains for younger (<65 years) and employed participants. The results of randomized intervention revealed higher median identified DRPs for older, unemployed or retired participants (*p* = 0.013; *p* = 0.018) in both groups. The most common manifested DRP was “untreated symptoms or indication,” with significantly higher number of identified potential DRPs in group A (*p* = 0.015). “Adverse drug event” was the most frequent potential DRP with higher frequency in group A. “Lack of cooperation of patient” was the leading reason for unresolved DRPs in both groups.

**Conclusion:**

Community pharmacists play an important role in identifying untreated conditions, adverse drug events and associated causes of DRPs related to psychotropic medicine use. Findings support integration of structured pharmaceutical care into community pharmacy practice to enhance patient safety through prevention of adverse drug events and medication safety surveillance.

## 1 Introduction

### 1.1 Mental health as a global burden

Mental health has been recognized as a public health priority at the global level, identified to be a crucial component of overall health and well-being (1). This recognition is especially important considering the increasing prevalence of mental disorders, its impact on various aspects of patients’ lives, including their families, broader communities and socio-economic implications (1). Mental disorders rank among the top 10 leading causes of global burden of diseases (GBD) with a 48.1% increase in estimated cases between 1990 and 2019 (2). According to the GBD Study 2019, the share of disability-adjusted life-years (DALYS) attributable to mental disorders increased from 3.1 to 4.9%, ranking them as the seventh leading cause of DALYs globally (2). These conditions are associated with lower quality of life (QoL), disability and premature mortality (3–8). Mental disorders can often have impact, but also are influenced by physical diseases (1). Compared with the general population, people with mental disorders experience approximately 14.7 years of potential life lost, primarily due coexisting physical diseases (8).

### 1.2 Mental health as a public health priority in Croatia

Given the high prevalence of mental disorders and their negative impact on QoL and mortality, including the potential long-term effects and significant use of healthcare resources, mental health has become one of the public health priorities in Croatia (9). In the pharmacological treatment of mental disorders, psycholeptics and psychoanaleptics are commonly used medicines. The annual and periodic reports on drug utilization in Croatia, published by the Agency for Medicinal Products and Medical Devices of Croatia (HALMED) indicate a steady increase in the use of these medicines (10–12). Drug utilization is described by the international unit – defined daily dose per 1,000 inhabitants per day (DDD/TID). DDD/TID estimates the proportion of residents using certain medicine, e.g., 10DDD/TID equals daily use of medicine in 1% of residents (10). In 2024, psycholeptic medicines ranked in fourth place in overall medicine utilization with a value of 113.60 DDD/TID (13). From 2018 to 2022, they had an annual increase of 1.8% in DDD/TID and a 2.4% increase in the financial costs (10). Over this period, anxiolytic medicines utilization is estimated to be double compared to other developed European countries. Conversely, antidepressants utilization in Croatia is rated to be twice as low, with a 4.4% annual utilization increase indicated by DDD/TID in the period from 2018 to 2022 (10). In Croatia, medicines with a pharmacological effect on the nervous system are also ranked high in overall reports of side-effects (14). Altogether, 59.8% of alerts from the Poison Control Center of the Institute for Medical Research and Occupational Health (IMI) are related to the mentioned medication group (14).

### 1.3 Drug related problems in mental health

Published data reveal various drug-related problems (DRPs) associated to the use of psychotropic medicines. The Pharmaceutical Care Network of Europe (PCNE) identifies a DRP as “an event or circumstance involving drug therapy that actually or potentially interferes with desired health outcomes” (15). That may relate to the effectiveness or safety of the drug use, presenting an important aspect of evidence-based pharmaceutical care. In terms of psychotropic medicines use, an important factor for patient safety is the manifestation of psychotropic drug–drug interactions (pDDI) and side-effects, especially among older patients. Negative impact of psychotropic medicine side-effects can possibly be observed on daily functioning and patients’ QoL, contributing to falls, hospitalization, but also mortality and morbidity (16–18). The effectiveness of pharmacotherapy can also be influenced by patients’ non-adherence to their treatment plans. According to Semahegn et al., non-adherence is observed in 49% of patients with major psychiatric disorders in community or facility-based settings (19). Identified contributors to non-adherence are sociodemographic factors, substance abuse, attitudes toward medication, perceived stigma, patients’ knowledge about illness or medication, side-effects, insufficient medication efficacy, treatment duration, complexity and co-morbidity, lack of social support and factors related to the health system (19). Kamaradova et al. showed in a cross-sectional study in the Czech Republic that self-stigma correlates negatively with adherence rates in patients with substance abuse disorders, schizophrenia, bipolar disorder, depressive disorders, anxiety disorders, and personality disorders. The results showed that self-stigmatization also contributes to voluntary discontinuation of medication (20).

### 1.4 Addressing clinical inertia

The recent research about possible improvement of patient safety in community-based mental healthcare settings, implies that management of risks can improve patients’ care and address patient and carer-centered safety concerns (21). That would mean to actively fight clinical inertia, which Philips et al. define as the failure to initiate or intensify therapy when indicated or the failure to act even though the problem has been recognized (22). Clinical inertia is usually researched in chronic diseases such as diabetes, dyslipidemia and hypertension. There is also increasing evidence of clinical inertia in psychiatry with studies on depression and bipolar disorder. For these diseases, there are clear guidelines and therapy goals that are necessary to overcome clinical inertia. It is recognized as the main cause of inadequate treatment of depression (23). Clinical inertia should be addressed on the individual and system level, with specific focus on stigma. Koschorke et al. reported that healthcare providers in primary care have expressed some willingness to help patients with mental illnesses, but they recognized the lack of knowledge about mental health problems and psychiatric medication (24). Their preference would be that mental healthcare should be delivered by specialists. Patients reported that they have limited expectations of support from primary care providers, therefore this research aim to explore ways to proactively approach the patients in community pharmacy setting. Some studies have shown that healthcare providers should be focused on equality, support, compassion, and confidentiality. Pharmacists could demonstrate all these attitudes and values, but they can also stand as the sustainable source of patients’ support, what was identified as a top priority from the stakeholders (25).

### 1.5 Community pharmacists and DRP management

Community pharmacists as the most accessible healthcare providers can play a very important role in identifying and addressing the DRPs related to the use of psychotropic medicines. Pharmacists have extensive knowledge but often lack the time, workforce capacity and adequate remuneration to deliver patient counseling (26, 27). Previous studies have indicated that “Pharmaceutical Care can significantly improve at least one domain of health-related QoL (HRQoL) across different medical conditions” (28). Pharmaceutical Care (PC) is defined by PCNE as a “pharmacist’s contribution to the care of individuals in order to optimize medicines use and improve health outcomes” (29). The benefits of PC have been studied in many contexts, particularly important when drugs with numerous side-effects and potential clinically significant drug–drug interactions are prescribed to the patient. These criteria are applicable to psychotropic medicines, among others, therefore the pharmacist’s intervention can make a significant difference. In addition, such interventions reduce costs in the healthcare system (30, 31).

Given these findings and the demonstrated need for structured pharmaceutical interventions and a more proactive approach by community pharmacists to address clinical inertia, it is necessary to further explore the real-life impact of pharmacist-provided care on patient-reported outcomes, particularly in primary care settings. This study aimed to evaluate the impact of structured PC on the identification and resolution of DRPs among patients using psycholeptics or psychoanaleptics, and to examine the relationship between sociodemographic factors and QoL in order to identify vulnerable groups.

## 2 Materials and methods

### 2.1 Participants, recruitment, and settings

This randomized controlled study was conducted in six community pharmacies located in Pula, Vodnjan, and Medulin (Istria County, Croatia). The inclusion criteria were adult patients (aged 18 years and older), using at least one psycholeptic or psychoanaleptic medicines and residence in Istrian County. Exclusion criteria included age below 18 years, hospitalization during study participation, or inability to participate for at least 3 months.

Participants were recruited during routine pharmacy conseling process in their respective community pharmacies after the informed consent was obtained.

The sample size was calculated using data on psycholeptic and psychoanaleptic utilization in Istria County in 2020 (32), and the estimated number of adult residents (33). The data on required drug utilization was obtained from HALMED for the purpose of this study. The sample size was calculated using a Creative Research Systems calculator (34) with a confidence level of 95% and a confidence interval of 10. Based on an estimated population of 21,443 residents in Istrian County who use psycholeptics or psychoanaleptics, the calculated sample size was 96 participants. Sampling was performed using stratified block randomization based on the location of the pharmacy. Using a computer random number generator, an allocation sequence was generated for each of the pharmacies (35–37).

**Figure 1:**
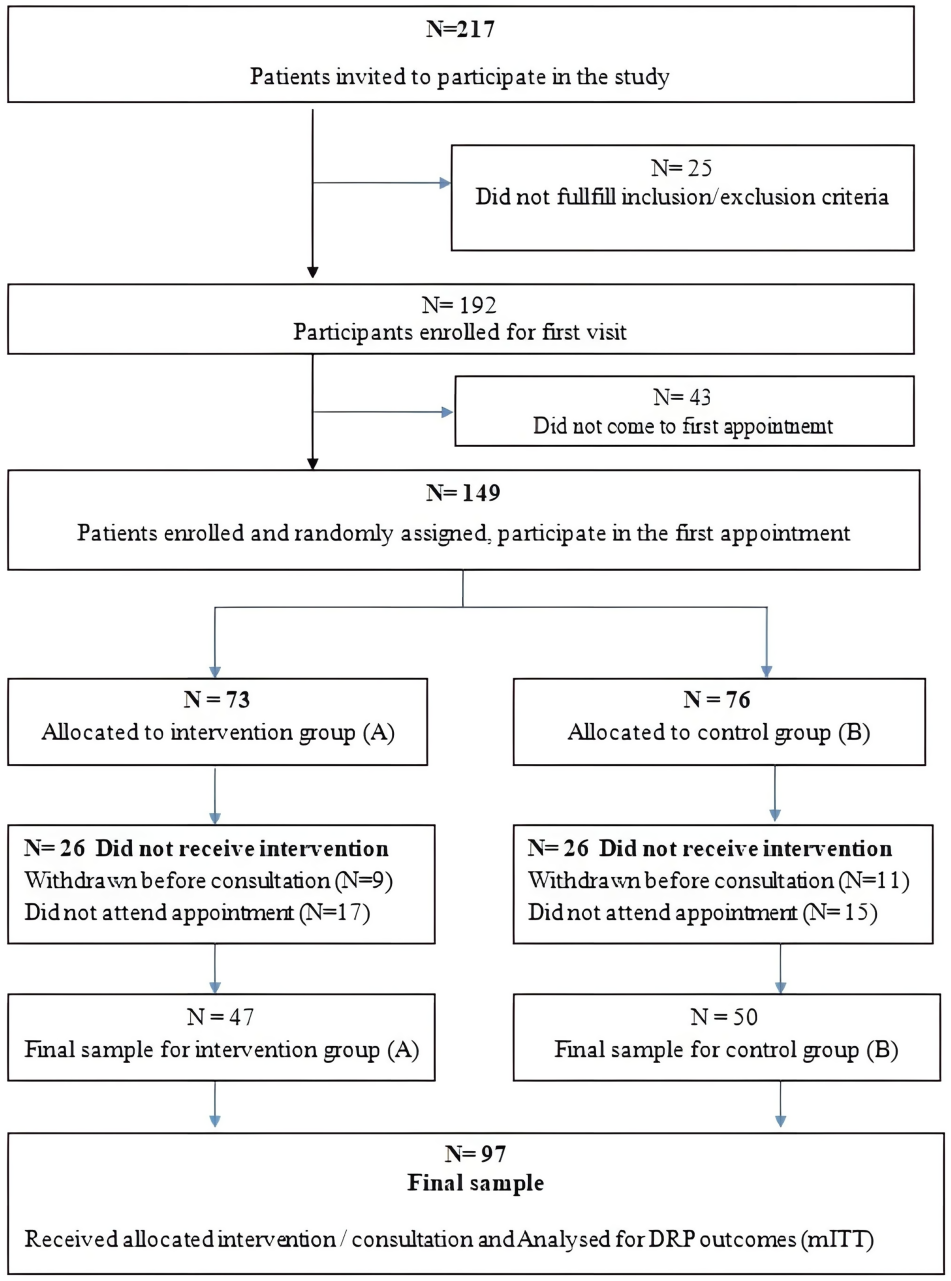
Participants enrolment diagram. *QoL was assessed at baseline only and therefore not included in follow-up or analysis stages of the flow diagram.

A modified intention-to-treat (mITT) approach was used. All participants who provided informed consent and attended at least one pharmaceutical care consultation were included in the analysis. Participants who withdrew before the first consultation were excluded because data were not available. Withdrawals were documented using the CONSORT flow chart (Figure 1). Given the real-world setting of community pharmacies and the sensitive nature of mental health care, loss to follow-up was expected. Therefore, the mITT approach was chosen to balance methodological rigor with feasibility while allowing for evaluation of the intervention among participants who entered the pharmaceutical care process.

### 2.2 Intervention

The study intervention involved the identification of DRPs and their underlying causes based on standardized protocol along with the pharmacist’s contribution in optimizing medicines use and resolving the identified DRPs. In both study groups, patients’ QoL was assessed before receiving pharmaceutical care. This study does not provide information on QoL changes following the intervention.

In group A, pharmacists’ interventions were based on standardized protocols for Medication Review (MR) and Medication Therapy Management (MTM) (38, 39). These services are commonly offered in various developed countries as part of standard pharmacy practice. In Croatia, however, these services are still in development and are not yet widely available. MR and MTM are structured evaluations of a patient’s pharmacotherapy aimed to optimize medicine use and improve treatment outcomes (38, 39). These processes involve identification of both potential and existing problems related to medicine use (e.g., medicine dosage, indication, methods of administration, patient adherence, potential drug interactions, patient’s understanding of the medication’s effects and associated warnings) and addressing these issues through pharmacist interventions. In this research, the pharmacist’s intervention occurred at the secondary level. This means that the intervention was based on information gathered from the patients, a list of medicines in use, and communication with the general practitioner when needed.

The pharmacists responsible for conducting the interventions in group A had previously participated in the MTM educational program from the Certified Training Program of the American Pharmacists’ Association (APhA) (39). This program aims to develop pharmacists’ knowledge, skills, and professional attitude necessary to provide effective PC.

Pharmacists who did not attend this program were involved in the data collection for the control group. Subjects allocated in the group B received PC based on common pharmacists´ practice in Croatia. Pharmacists who provided common pharmaceutical care did not follow specific protocol. All pharmacists who participated in the data collection, used same questionnaires for baseline QoL measurement and documentation of DRPs.

### 2.3 Questionnaires and coding

To assess the baseline patient’s QoL, the World Health Organization Quality of Life-BREF (WHOQOL-BREF) questionnaire was used (40). Research on its psychometric features indicates that the WHOQOL-BREF is a reliable and valid instrument, with a correlation to the WHOQOL-100 of approximately 0.89 (41). The WHOQOL-BREF questionnaire comprises 26 questions organized into 4 QoL domains: Physical health, Psychological health, Social relationships and the Individual’s environment. Each question is rated on a Likert scale from 1 (worst) to 5 (best), meaning that a higher score reflects a higher QoL (40).

Participants completed the WHOQOL-BREF questionnaire independently or, if needed, with assistance from a pharmacist. The domain score was calculated by taking the mean value of the answers to the relevant questions, which is then multiplied by 4 to make the scores comparable to the ones used in the WHOQOL-100 questionnaire (41).

For the documentation of the DRPs and pharmacist’s interventions, the validated PCNE DRP ClassificationVersion 9.1 was used by all pharmacists in both study groups (42). This classification facilitates the documentation of identified DRPs, their causes, then pharmacist’s interventions to address the causes, the level of patient’s acceptance of proposed interventions, and the status of each identified DRP after the pharmacists’ intervention. The PCNE DRP Classification is structured within primary domains and subdomains. Each domain and subdomain has a specific code, which facilitates the processing of collected data. For each identified DRP, the PCNE recommends documenting up to three identified causes and planned interventions.

Sociodemographic data was collected and organized into specific categories, including gender, age, employment, marital status and level of education.

### 2.4 Statistical analysis

The data distribution was tested using either the Kolmogorov–Smirnov test or the Shapiro–Wilk test, depending on the number of subjects per group. Since all compared data showed a skewed distribution, non-parametric tests were used for analysis. The Chi-square test for homogeneity and Fisher’s exact test were used to assess whether the distributions of categorical variables differed between groups A and B. The Mann–Whitney test was employed for comparing two independent groups, A and B.

Baseline QoL scores were compared between patients with different numbers of DRPs using the Kruskal-Wallis test, followed by the Mann–Whitney test as a *post hoc* analysis. The relationship between DRPs and baseline QoL measurements was analyzed using Spearman’s correlation. Data are presented as the median and interquartile range (25th–75th percentile) for independent data, and as median of difference for dependent data with interquartile range. Categorical variables are expressed as relative or absolute frequencies. All analyses were performed using SPSS 27.0 (SPSS Inc., Chicago, United States). A two-tailed *p*-value ≤ 0.05 was considered statistically significant.

### 2.5 Ethical consideration

The study was conducted in accordance with the Declaration of Helsinki and approved by two ethics committees. Written informed consent was obtained from all participants prior to study inclusion. Participation in the research was voluntary. The research methodology was based on ethical and bioethical principles: autonomy, justice, beneficence, and non-maleficence. Personal and medical data were collected in compliance with bioethical standards.

## 3 Results

Ninety seven participants were assessed in the study. The intervention (A) and control (B) groups were homogenous in terms of sociodemographic characteristics (Table 1).

**Table 1:** Allocation and sociodemographic characteristics.

Sociodemographic characteristic		A group N (%)	B group N (%)	Total N (%)	*p*
Gender	Male	13 (27.7)	18 (36.0)	97 (100)	0.379
	Female	34 (72.3)	32 (64.0)		
Age	< 65 years	24 (51.06)	28 (56.0)	97 (100)	0.626
	≥ 65 years	23 (48.94)	22(44.0)		
Working status	Employed	18 (38.3)	14 (28.0)	97 (100)	0.281
	Unemployed or in retirement	29 (61.7)	36 (72.0)		
Marital status	Single, divorced or widow(−er)	16 (34.0)	23 (46.0)	97 (100)	0.230
	In marriage or consensual union	31 (66.0)	27 (54.0)		
Level of education	Unfinished school or primary school	6 (13.6)	4 (8.3)	92 (94.9)^1^	0.677
	Secondary vocational school	13 (29.5)	12 (25.0)		
	High school	11 (25.0)	17 (35.4)		
	Higher vocational school	14 (31.8)	15 (31.3)		

^1^Missing data for five participants regarding their level of education.

The proportion of patients age 65 or older was 48.94% in group A and 44% in group B 44%, *p* = 0.626.

In both groups the majority of participants were female (group A 72.3%; group B 64.0%, *p* = 0.379). Most participants were unemployed or retired (group A 61.7%; group B 72.0%, *p* = 0.281).

### 3.1 Relation of sociodemographic data and quality of life

Data normality was tested using Shapiro–Wilk test revealing non-normal distribution across groups. Comparisons of baseline QoL measurements between groups using the Mann–Whitney U test showed no statistical significance difference in Physical, Psychological or Social domains, but a significantly higher scores for the Environment domail in group A (*p* = 0.041) (Table 2).

**Table 2:** Comparison of the QoL domains between groups.

QoL domain (0–100)	A group Median (IQR) Min-max	B group Median (IQR) Min-max	*p*
Physical health	63 (50.0–78.0) 0–100	56 (38.0–73.5) 0–94	0.265
Psychological health	69 (50.0–81.0) 19–94	69 (56.0–81.0) 0–88	0.855
Social relationships	56 (44.0–78.0) 0–100	56 (45.5–69.0) 0–100	0.298
Individual’s environment	69 (56.0–81.0) 0–100	63 (50.0–75.0) 0–100	**0.041**

IQR, interquartile range. Bold values indicate statistically significant result.

The analysis of baseline QoL scores and sociodemographic data relationship releved higher scores of Physical health (*p* = 0.018), Social interaction (*p* < 0.001) and Individual’s environment domains in participants who were employed (Table 3). In terms of the age influence, participants younger than 65 years had higher score of Physical health (*p* = 0.018) and Social interacion (*p* < 0.001) (Table 4). Gender and marital status differences had no statistical relevence.

**Table 3:** Relationship of the QoL domains and working status for all participants.

**QoL domain** **(0–100)**	**Employed** **Median (IQR)** **Min-max**	**Unemployed or in retirement** **Median (IQR)** **Min-max**	***p***
Physical health	63 (56–81) 13–100	56 (38–69) 0–94	**0.018**
Psychological health	69 (56–81) 19–88	69 (56–81) 0–94	0.849
Social relationships	72 (56–81) 19–100	56 (31–69) 0–100	**< 0.001**
Individual’s environment	72 (63–81) 25–100	63 (44–75) 25–100	**0.019**

IQR, interquartile range. Bold values indicate statistically significant result.

**Table 4:** Relationship of the QoL domains and age for all participants.

QoL domain (0–100)	Age < 65 years Median (IQR) Min-max	Age ≥ 65 years Median (IQR) Min-max	*p*
Physical health	63 (56–75.5) 0–100	50 (25–69) 0–94	**0.018**
Psychological health	69 (56–81) 19–88	69 (56–81) 0–94	0.315
Social relationships	69 (56–81) 0–100	50 (6–56) 0–100	**< 0.001**
Individual’s environment	69 (54.5–81) 0–100	63 (38–75) 0–100	0.116

IQR, interquartile range. Bold values indicate statistically significant result.

### 3.2 Drug-related problems in patients using psychotropic medicines

#### 3.2.1 Relation of sociodemographic data, drug-related problems, and psychotropic medicine

The mean number of psychotropic medicines in use per participant was similar in both groups; the median (25th–75th percentile) in group A was 1 (1–2) and in group B it was 2 (1–2) (*p* = 0.162). However, unemployed or retired participants showed a higher median number of DRPs (*p* = 0.018), despite no significant difference in the total number of psychotropic medicines in use (*p* = 0.076).

In addition, age had a significant influence on the use of psychotropic medicines (*p* = 0.009). Fifty-six percent of participants younger than 65 years used at least two psychotropic medicines [median 2 (1–2)], compared to the older group, where all participants used at least one psychotropic medicine [median 1 (1–2)] (Table 5). Although there was no significant difference in the median number of DRPs between the two age groups [younger group median 1 (0.25–2), older group median 1 (1–2), *p* = 0.107] there was detected significance in the presence of DRPs in older participants. Only 7% of participants older than 65 years had no identified DRP compared to 26% of younger participants with no DRP (*p* = 0.013) (Table 6).

**Table 5:** Number of psychotropic medicines in use per participant compared to age.

Psychotropic medicine per participant N	N < 65 years	N ≥ 65 years
1	23	30
2	17	11
3	10	3
5	2	0
Total*	52	44

*For one participant older than 65 years the complete medication record was not available.

**Table 6:** Number of DRPs per participant compared to age.

DRPs per participant N	N < 65 years	N ≥ 65 years
0	13	3
1	21	24
2	13	13
3	0	4
4	2	0
6	1	0
Total*	50	44

*Two participants under 65 years and one participant over 65 years did not want counseling from pharmacists, but were willing to complete the QoL questionnaire.

Gender, marital status and level of education showed no statistical differences when it comes to the total number of identified DRPs per participant or total number of psychotropic medicines in use.

#### 3.2.2 Distribution of identified drug-related problems

Identified DRPs were classified as manifested or potential. The number of identified potential DRPs was higher in group A (A vs. B, 40 vs. 26, *p* = 0.015) with no difference in the number of identified manifested DRPs between groups (A vs. B, 28 vs. 26, *p* = 0.700).

The most prevalent manifested DRP in both groups was “untreated symptoms or indication” (% of participants in A vs. B, 21.3 vs. 22.0, *p* = 0.884). The second most frequent DRP in the category of manifested DRPs was “adverse drug event (possibly) occurring” (% of participants in A vs. B, 14.9 vs. 16.0, *p* = 0.880).

**Figure 2:**
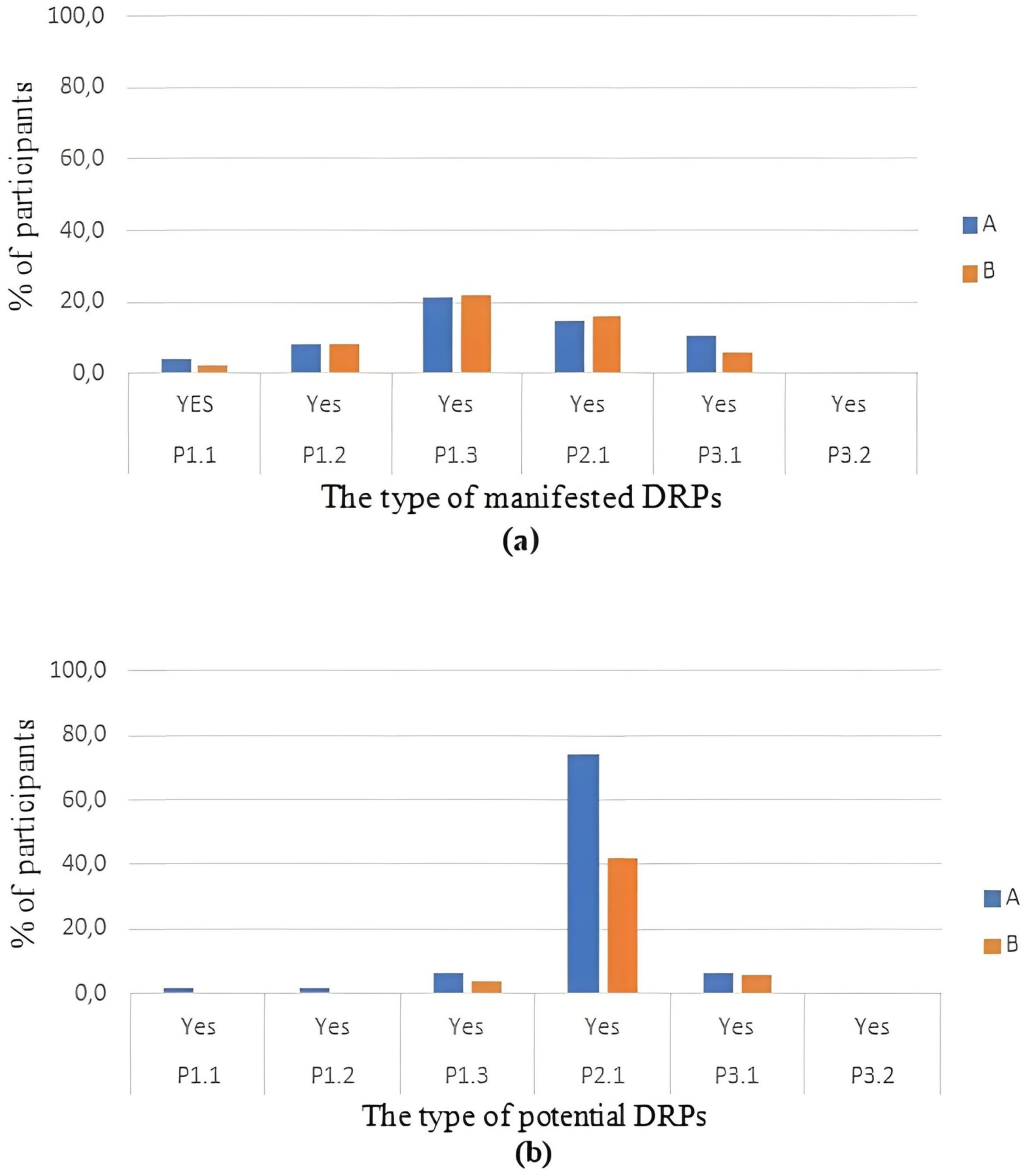
Percentage of participants in groups A and B with: **(a)** manifested problems; **(b)** potential problems. The type of DRPs are coded according to PCNE DRP Classification Version 9.1: P1.1, No effect of drug treatment despite correct use; P1.2, Effect of drug treatment not optimal; P1.3, Untreated symptoms or indication; P2.1, Adverse drug event (possibly) occurring; P3.1,Unnecessary drug-treatment; P3.2, Unclear problem/complaint.

Furthermore, data analysis indicates “adverse drug event (possibly) occurring” as the most frequent type of potential DRP (% of participants in A vs. B, 74.5 vs. 42.0, *p* = 0.001) with higher frequency in group A (Figure 2).

#### 3.2.3 Identified causes of drug-related problems

According to PCNE guidelines, up to maximum three causes can be indicated for each identified DRP. Both groups had a similar distribution of identified causes for manifested and potential DRPs.

The most frequent cause for manifested DRPs was “duration of treatment too long” (% of all causes for manifested DRPs in A vs. B, 29.51 vs. 29.31, *p* = 0.981), with “no or incomplete drug treatment in spite of existing indication” (% of all causes for manifested DRPs in A vs. B, 21.31 vs. 20.69, *p* = 0.934) and “no or inappropriate outcome monitoring” (% of all causes for manifested DRPs in A vs. B, 14.75 vs. 18.97, *p* = 0.539) as the second and third most common causes of manifested DRPs (Table 7).

**Table 7:** Distribution of identified causes for manifested DRPs.

Causes^+^	A group N(%)	B group N(%)	P*
Inappropriate drug according to guidelines/formulary	8(13.11)	7(12.07)	0.864
No indication for drug	1(1.64)	1(1.72)	0.971
Inappropriate duplication of therapeutic group or active ingredient	/	3(5.17)	/
No or incomplete drug treatment in spite of existing indication	13(21.31)	12(20.69)	0.934
Drug dose of a single active ingredient too high	1(1.64)	/	/
Duration of treatment too long	18(29.51)	17(29.31)	0.981
Patient intentionally uses/takes less drug than prescribed or does not take the drug at all for whatever reason	1(1.64)	/	/
Patient uses/takes more drug than prescribed	5(8.2)	1(1.72)	0.139
Patient decides to use unnecessary drug	1(1.64)	2(3.45)	0.529
No or inappropriate outcome monitoring	9(14.75)	11(18.97)	0.539
Other cause	4(6.56)	4(6.9)	0.941
Total	61(100)	58(100)	

*Chi-square test: comparison of each cause vs. others. +Identified causes according to PCNE DRP Classification Version 9.1.

For potential DRPs the most frequently identified cause was “inappropriate drug according to guidelines/formulary” (% of all causes for potential DRPs in A vs. B, 30.14 vs. 34.29, *p* = 0.664). “Duration of treatment too long” (% of all causes for potential DRPs in A vs. B, 21.92 vs. 20, *p* = 0.820) and “other causes” (% of all causes for potential DRPs in A vs. B, 13.69 vs. 14.28, *p* = 0.739) were second and third frequent causes (Table 8).

**Table 8:** Distribution of identified causes for potential DRPs.

Causes^+^	A group N(%)	B group N(%)	P*
Inappropriate drug according to guidelines/formulary	22(30.14)	12(34.29)	0.664
No indication for drug	/	1(2.86)	/
Inappropriate combination of drugs, or drugs and herbal medications, or drugs and dietary supplements	1(1.37)	/	/
Inappropriate duplication of therapeutic group or active ingredient	6(8.22)	3(8.57)	0.950
No or incomplete drug treatment in spite of existing indication	5(6.85)	2(5.71)	0.823
Too many different drugs/active ingredients prescribed for indication	1(1.37)	1(2.86)	0.606
Drug dose of a single active ingredient too high	1(1.37)	/	/
Duration of treatment too long	16(21.92)	7(20)	0.820
Patient intentionally uses/takes less drug than prescribed or does not take the drug at all for whatever reason	/	/	
Patient uses/takes more drug than prescribed	7(9.59)	1(2.86)	0.211
Patient decides to use unnecessary drug	/	/	/
No or inappropriate outcome monitoring	4(5.48)	3(8.57)	0.541
Other cause	10(13.69)	5(14.28)	0.739
Total	73(100)	35(100)	

*Chi-square test: comparison of each cause vs. others. + Identified causes according to PCNE DRP Classification Version 9.1.

#### 3.2.4 The outcomes of the provision of pharmaceutical care

After PC was provided, in the category of manifested problems, the outcomes of DRPs were described by pharmacists as “totally solved” in 8.5% (group A) and 6% (group B) with “partially solved” in 6.4% (group A) and 6% (group B).

In the category of potential DRPs, “totally solved” DRPs were observed in 8.5% (group A) and 2% (group B) with “partially solved” in 2.1% (group A) and 6% (group B) of participants.

**Figure 3:**
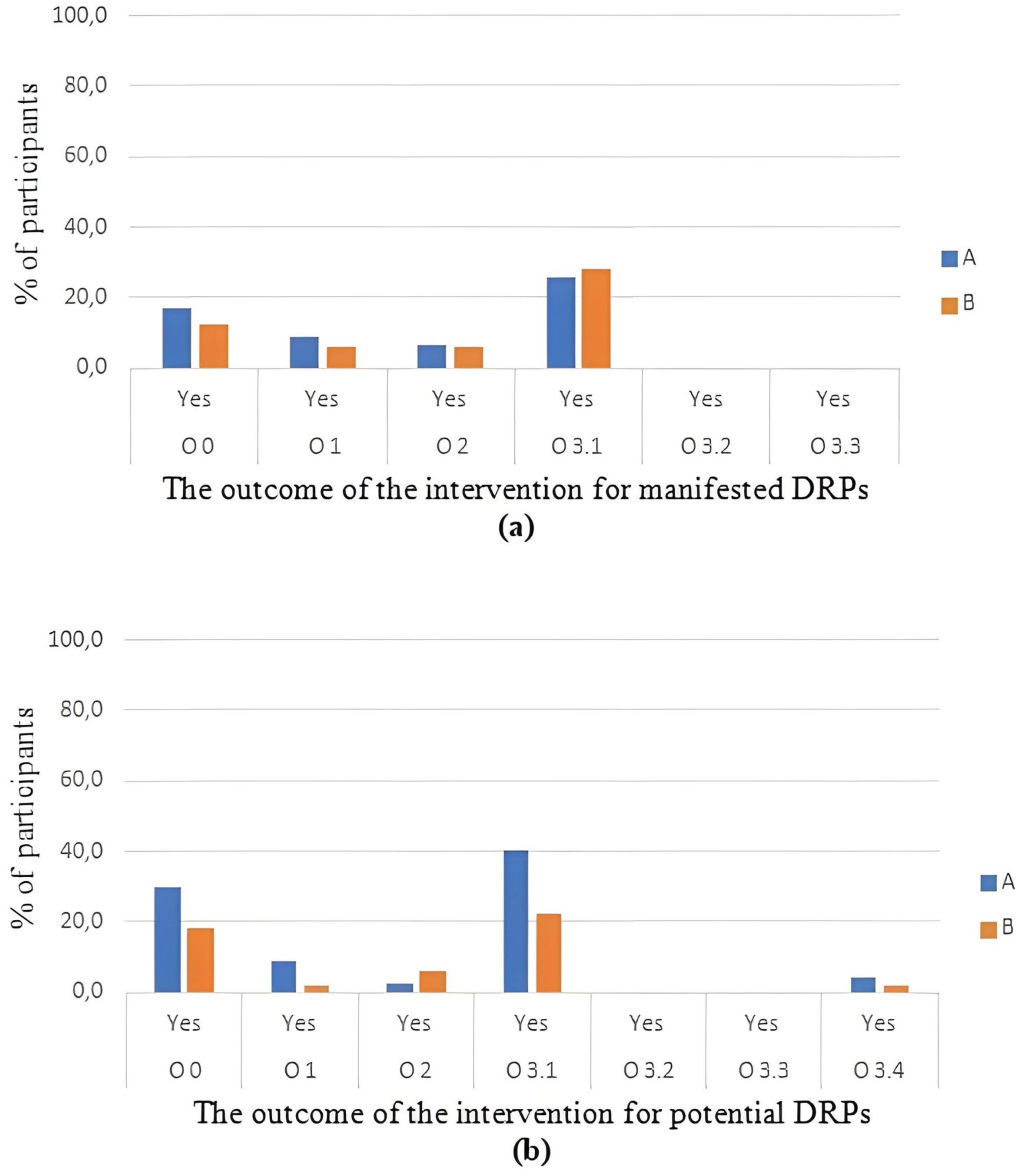
Percentage of participants in groups A and B in relation to the outcome of the intervention for: **(a)** manifested problems and **(b)** potential problems. O0, Problem status unknown; O1, Problem totally solved; O2, Problem partially solved; O3, Problem not solved; O3.1, Lack of cooperation of patient; O3.2, Lack of cooperation of prescriber; O3.3, Intervention not effective; O3.4, No need or possibility to solve problem.

When the status of potential and manifested DRPs was described as “not solved” after the pharmacists´ intervention, the most common reason, described by pharmacists, was “lack of cooperation of patient” in both groups (manifested DRPs- group A 25.5%, group B 28% of participants, *p* = 0.783; potential DRPs - group A 40.4%, group B 22% of participants, *p* = 0.059) (Figure 3).

### 3.3 Correlation of drug-related problems and quality of life

Spearman’s rho analysis of DRPs and baseline measurement of QoL has shown a negative correlation, but with no statistical significance. Spearman’s rho and *p*-values for the domains were: Physical Health (r-0.105, *p* = 0.308), Psychological Health (r-0.085, *p* = 0.412), Social Interaction (r-0.189, *p* = 0.065), and Individual’s Environment (r-0.103, *p* = 0.316).

Although no significant negative correlation was observed between DRPs and baseline QoL, we analyzed the differences in baseline QoL among patients with different numbers of DRPs (ranging from 0 to 3 or more). A difference in baseline QoL scores was observed only in two domains, Physical health and Social interactions. Although baseline QoL domain scores were the lowest in participants with three or more identified DRPs, statistical significance was detected between participants with 1 DRP or non-DRP detected (Table 9).

**Table 9:** Correlation of drug-related problems and quality of life.

QoL domain (0–100)	DRP0 Median (IQR) N16	DRP1 Median (IQR) N45	DRP2 Median (IQR) N26	DRP ≥3 Median (IQR) N9	*p*
Physical health	69 (56–81)	56 (25–69)*	63 (51.1–69)	38 (25–75)	**0.045**
Psychological health	72 (61.3–81)	69 (50–81)	63 (56–81)	63 (56–81)	0.428
Social interactions	75 (65.8–81)	56 (44–69)†	56 (45.5–69)	56 (6–69)	**0.011**
Individual’s environment	69 (63–84.3)	63 (38–81)	69 (63–79.5)	50 (25–69)	0.094

IQR, interquartile range; N, number of patients with non-DRP detected, or with 1, 2, 3, and more. *Significant difference compared to non- DRP group (*p* = 0.045). †Significant difference compared to non- DRP group (*p* = 0.011). Total N (96)—one patient did not want pharmaceutical care and identification of DRPs to be performed, but was willing to complete the self-assessed QoL questionnaire. Bold values indicate statistically significant result.

## 4 Discussion

### 4.1 The importance of structured and comprehensive pharmaceutical care in mental health

Participants included in this study did not statistically differ between groups regarding sociodemographic characteristics, number of psychotropic medicines in use or in the baseline measurement of QoL level, except for satisfaction of individual’s environment. The similarity of the study groups suggests that study findings are relevant to the intervention being studied.

Results from this study point to the importance of structured and comprehensive pharmaceutical care in mental health. Although the number of identified manifested DRPs was similar in the intervention and control groups, additional importance is seen in the significantly higher detection rate of potential DRPs in the intervention group (*p* = 0.015). In contrast, the observed modest rates of PC outcomes regarding totally or partially solved DRPs, in both study groups, highlight the challenges that remain in the resolution of DRPs. This study has confirmed that “lack of patient cooperation” is one of the main reasons why DRPs are not resolved. These findings emphasize the potential of PC in preventing DRPs, while also pointing out the need for improved patient adherence to mental health treatments.

Lower scores were identified in the Physical and Social Interaction domains in the baseline measurement of QoL for participants older than 65 years, unemployed or in retirement. Recognizing these factors can help in the early identification of patients using psychotropic medicines who are at a higher risk for reduced QoL. Additionally, these two sociodemographic factors were associated with a greater presence of identified DRPs.

These findings highlight the crucial role that pharmacists can play in primary public health interventions aimed at preventing DRPs before they occur, as well as reducing associated risk factors. This is particularly important for older patients and those who are unemployed or retired.

On a global scale, mental disorders are the leading cause of years lived with disability (43). Limited access to mental health services and psychotropic medicines leads to their inefficient use and significant barriers to providing proper treatment of mental disorders (44). This study, conducted in community pharmacies, enhanced accessibility to mental health services provided by pharmacists, as well as promotion of mental well-being and stigma reduction.

An additional issue to consider in the treatment of mental disorders is the appropriate use of psychotropic medicines. It is estimated that “more than 50% of all medicines are prescribed, dispensed or sold inappropriately, and half of all patients fail to take them correctly” (45) (p. 46). The “lack of patient cooperation,” observed in this study, is important to consider in terms of irrational medicine use, which can lead to negative health outcomes and significant waste of healthcare resources (45).

### 4.2 Adverse drug reactions of psychotropic medicines

The use of psychotropic medicines is associated with various DRPs, which require careful monitoring to ensure treatment effectiveness and patient safety. In the literature, psychotropic medicines are known for their extensive adverse drug reactions and high potential for clinically significant drug–drug interactions (46). In this study, when additional questions were asked during the PC process, well-known risks for the development of ADRs related to the use of psychotropic medicines, were recognized and discovered. Using structured questionnaires is helpful in identifying manifested and/or potential ADRs.

As highlighted by the WHO, coordinated action across multiple levels of the healthcare system is needed to achieve appropriate use of psychotropic medicines (45). One of the advantages of this study was increased awareness by patients that ADRs can be treated and reported, as well as prevented in cooperation with their pharmacists and other healthcare providers.

Published data regarding pharmacist-led interventions point to the different benefits for patients and the healthcare system (46, 47). Identification, resolution and prevention of DRPs led by clinical or community pharmacists can help to improve clinical, economic and humanistic outcomes in patients suffering from mental disorders (46, 47). The manifestation and non-resolution of developed ADRs can be an important obstacle in achieving mentioned outcomes.

In this study, adverse drug reactions are recognized as the most common potential DRPs and ranked as the second most frequent in the category of manifested problems. This aligns with findings from Jayakumar et al. (48), which indicate that over half of inpatients in the psychiatry department presented DRPs, with adverse drug reactions and potential drug–drug interactions being the most commonly identified DRPs. The assessment of potential drug interactions of psycholeptic medicines and antidepressants conducted by Marović et al. (49), describes the increased risk of CNS depression and risk of QTc interval prolongation as the most common consequences of the determined potential psychotropic drug interactions in outpatient settings. This affirms the importance of PC in prevention, detection and resolution of ADRs provided during this study.

### 4.3 Importance of early detection of untreated conditions and related causes in patients using psychotropic medicines

The analysis of identified DRPs in patients using psychotropic medicines revealed “untreated symptom or indication” as the most common manifested DRP in both of the study groups (Figure 2), which is one of the main findings of this study.

Three main causes of identified manifested DRPs were “duration of treatment too long,” “no or incomplete drug treatment” and “no or inappropriate outcome monitoring” with “inappropriate drug according to guidelines/formulary” as the most common cause of identified potential DRPs.

This is important to mention considering the role of pharmacists in the early detection of mental health complication, screening for signs and symptoms of mental disorders or their deterioration, referral of patients to other healthcare specialists, promotion of proper and timely treatment and pharmacotherapy optimization.

From previous studies we know that recognizing and being able to differentiate anxiety and depression from other underlying medical illnesses (e.g., cardiac disorders; withdrawal from alcohol, caffeine, or nicotine; endocrine disorders; sleeping disorders) can be difficult due to similar symptoms being explained by the patients (50). Potentially undiagnosed depression and anxiety, chronic sleeping problems and untreated co-morbid diseases (e.g., dyslipidemia, high blood pressure, and weight gain) are some of the examples observed during conducting the study and providing care to patients.

The relationship between specific illnesses and medical treatments and their impact on QoL is an area of growing interest for researchers. In many studies, self-reported QoL is commonly used as a measure to assess treatment outcomes. Reduced level of QoL in people suffering from mental disorders is evident in published data (3–7).

In this study, correlation between identified DRPs and baseline QoL pointed to a statistically significant difference in the Physical health and Social interactions domains between participants with one DRP or no DRP detected. The non-significant result related to the baseline QoL and higher number of DRPs per patient, can be explained by a smaller number of participants with three or more DRPs.

Nevertheless, these findings highlight the significance of early detection and resolution of DRPs and their causes to enhance patients’ QoL.

### 4.4 Non-adherence related to the use of psychotropic medicines

Another significant problem related to the use of psychotropic medicines is non-adherence. In this study the lack of patient cooperation has been identified as a significant obstacle in the resolution of DRPs. The reasons for that outcome have not been collected from participants during the study, but this behavior was expected, as it is known from the literature, that psychotropic treatment non-adherence among patients with psychiatric disorders was quite common.

Recent systematic review and meta-analyses have shown that non-adherence was 46% and even higher among patients with major depressive disorders. Sociodemographic and other patient-related factors, such as negative attitude toward the treatment, poor social support, self-stigma, and especially drug-related factors (aOR = 1.42, I^2^ = 64.16%) were the predictors (51). The WHO’s Mental Health Survey Initiative indicates that the dropout rate for outpatient mental health care is high, 30% in high-income countries and 45% in low/middle-income countries. Dropout is most likely to occur during the first two visits (52).

If non-adherence is not detected or resolved, it can lead to symptom worsening, relapses, hospitalization, and an increased risk of suicide. To overcome that barrier, the authors believe that an effective solution lies in improved collaboration among pharmacists, general practitioners, and psychiatrists. The study by Shi et al. (53) found an 83.46% success rate after pharmacists’ interventions in a hospital setting. Studies on pharmacists’ impact on medication adherence in patients with mental disorders showed improvements in adherence across community pharmacies, hospitals, and interdisciplinary mental health clinics (54). The effectiveness of pharmacists’ interventions has been influenced by healthcare settings (55). Future thorough analysis of specific DRPs and direct pharmacists’ interventions would enhance the understanding of the role of pharmacists in the care of patients with mental disorders.

### 4.5 Limitations of the study

There are several limitations of the study. Potential confounding factors, such as comorbidities, social support, or the severity of mental illness, could not be fully monitored due to the real-world setting and sample size. However, the researchers attempted to reduce their influence by ensuring homogeneity between the groups in key sociodemographic characteristics, using validated instruments and conducting within-group analyses. Patients with mental health often have problems with adherence, and potentially with follow-up visits. Despite the initial identification of a larger eligible sample, a notable number of individuals declined to participate (Figure 1). Given the existing evidence of high dropout rates in outpatient mental health care, we expected to find similar trends in our study (56). The specific nature of mental health issues often include social fobia, as a limitation to accept interventions of healthcare professionals (57). The sensitivity of invididuals’ mental health should be taken into consideration (58). Recognizing that good social contact is among the most effective strategies for addressing stigma-related attitudes, this study was conceived as a response to that need (59).

The heterogeneity of the psychotropic medicines included in the study, type of the present mental disorders and associated co-morbidities, can be an influencing factor on the number of detected DRPs, their presentations, estimated level of complexity and severity. Furthermore, this study did not assess the correlation between prescription rates and factors such as age, gender, socioeconomic status, or geographic location, which can influence various mental health needs.

Findings from a large cross-sectional, population-based study reveal variations in psychotropic medication use across Europe based on gender and age. The study demonstrated a positive correlation between age and prescription rates, with the highest rates observed among individuals aged 65 and older (60). Gender-based differences in psychotropic prescribing patterns have also been documented. Several studies have found that females are prescribed psychotropic medicines more frequently than males, with the notable exception of antipsychotics. Women are more likely to consult general practitioners for mental health concerns, whereas men more often seek help from mental health specialists. Since general practitioners are more inclined to prescribe psychotropic medicines, while specialists tend to recommend alternative treatments such as psychotherapy, this may partially explain the observed gender disparity in prescribing rates (60, 61).

Socioeconomic status (SES) is another factor frequently examined in the literature. Individuals with low to moderate SES tend to have higher rates of psychotropic medication prescriptions. Moreover, mental health disorders are often associated with lower levels of educational attainment and unemployment (61, 62). Geographical location also influences prescription rates. Previous studies have reported differences between urban and rural areas, with lower prescription rates observed in rural settings. This disparity may be attributed to reduced access to mental health professionals in rural areas, as well as a greater reluctance to seek help due to stigma (61, 62).

Another important limitation relates to variability in pharmacists’ knowledge, experience, and confidence in managing psychotropic pharmacotherapy, which was not formally assessed in this study. In developed countries, there is a variety of extended and advanced pharmacy services for mental health care (63). In the countries of south-eastern Europe, there is neither specific training nor specialization programs for pharmacists in the field of mental health. Challenges such as emotional discomfort, limited training in communication, and unpredictable patient behavior are documented barriers to effective mental health care in pharmacy settings (64).

### 4.6 Advantages of the study

To our knowledge, this is the first study conducted in community pharmacy settings in Croatia that evaluates QoL and the impact of PC in patients using psychotropic medicines and addresses clinical inertia. The intervention was embedded in routine professional activities, which increases its real-world applicability. Collaboration with general practitioners was established, though further improvements in interdisciplinary cooperation are needed. The role of community pharmacists in mental health care is evolving, and new services are emerging (64). However, there is a small number of well-documented models that could be implemented worldwide compared to other non-communicable diseases. Frequently cited barriers include organizational culture, limited interprofessional collaboration, mental health stigma (both in patients and healthcare providers), insufficient pharmacist training, and lack of sustainable remuneration models (65, 66).

This study presents a good practice model where most of these barriers were addressed, except for remuneration. It illustrates how a trained pharmacist collaborating with physicians can consistently deliver pharmaceutical care using validated tools and serves as an example to impact clinical inertia. The most frequently observed DRPs in this study may serve as a foundation for developing standardized protocols for mental health counseling in pharmacy practice.

A literature search using the keywords “community pharmacist,” “psychotropic drug,” “intervention,” and “drug-related problem (DRP)” reveals the existence of 5 studies. None of those studies were related to community pharmacy services, but either inpatient hospital intervention or tele-psychiatric clinical services (53, 67–70). The authors believe that this example can serve as evidence that important interventions in mental health can also be made in community pharmacies, not just in hospital settings or specialized clinics, with the contribution to public health.

Future studies should aim to investigate whether pharmacists’ interventions led to improvement of QoL in patients with mental health issues. Larger and more diverse samples could be included, and new investigation objectives could be to evaluate pharmacists’ competencies, and explore reasons behind patient non-cooperation in resolving DRPs.

## 5 Conclusion

Identifying “untreated symptoms or indications” and “adverse drug event” as the most frequent DRPs in this study, highlights the need for community pharmacists to actively participate in the care of patients using psychotropic medicines. The study presents how community pharmacist can involve in reducing the clinical inertia in mental health care. The contribution of PC in the prevention of different categories of DRPs can be of major importance for patient safety and treatment outcomes as seen in the detection of potential DRPs.

Although in this study, a higher proportion of participants had unresolved DRPs compared to those with totally or partially solved DRPs, community pharmacists play an important role in the early recognition of untreated conditions, adverse drug reactions and associated causes of DRPs in patients using psychotropic medicines. This study emphasize pharmacists’ role in preventing DRPs and related risks—especially among older, unemployed, or retired patients. Detected lack of patient cooperation as a major identified contributor of not solved DRPs can serve as a new objective for future public mental health initiatives.

The study findings support integration of structured pharmaceutical care into community pharmacy practice to enhance patient safety through prevention of adverse drug events and medication safety surveillance.

## Data Availability

The raw data supporting the conclusions of this article will be made available by the authors, without undue reservation.
